# Short-term effect of low-dose colchicine on inflammatory biomarkers, lipids, blood count and renal function in chronic coronary artery disease and elevated high-sensitivity C-reactive protein

**DOI:** 10.1371/journal.pone.0237665

**Published:** 2020-08-31

**Authors:** Aernoud T. L. Fiolet, Max J. M. Silvis, Tjerk S. J. Opstal, Willem A. Bax, Frans A. L. van der Horst, Arend Mosterd, Dominique de Kleijn, Jan H. Cornel

**Affiliations:** 1 Department of Cardiology, University Medical Center Utrecht, Utrecht, The Netherlands; 2 Dutch Network for Cardiovascular Research, Utrecht, The Netherlands; 3 The Netherlands Heart Institute, Utrecht, The Netherlands; 4 Department of Cardiology, Northwest Clinics, Alkmaar, The Netherlands; 5 Department of Internal Medicine, Northwest Clinics, Alkmaar, The Netherlands; 6 Department of Clinical Chemistry, Haga Zorggroep, Delft, The Netherlands; 7 Department of Cardiology, Meander Medisch Centrum, Amersfoort, The Netherlands; 8 Julius Center for Health Sciences and Primary Care, University Medical Center Utrecht, Utrecht, The Netherlands; 9 Department of Vascular Surgery, University Medical Center Utrecht, Utrecht, The Netherlands; 10 Department of Cardiology, Radboud University Medical Center, Nijmegen, The Netherlands; Universita degli Studi di Milano, ITALY

## Abstract

**Aims:**

Inflammation plays a pivotal role in atherothrombosis. Colchicine is an anti-inflammatory drug that may attenuate this process. Cardiovascular protective effects of anti-inflammatory drugs, however, seem to be limited to patients with a biochemical response. We therefore investigated whether short-term exposure to colchicine reduced inflammatory markers and whether additional laboratory changes occur in patients with chronic coronary artery disease.

**Methods & results:**

In 138 consecutive patients with chronic coronary artery disease and a high sensitivity C-reactive Protein (hs-CRP) ≥ 2 mg/L, inflammatory markers, lipids, haematologic parameters and renal function were measured at baseline and after 30 days exposure to colchicine 0.5mg once daily. Hs-CRP decreased from baseline 4.40 mg/L (interquartile range [IQR] 2.83–6.99 mg/L) to 2.33 mg/L (IQR 1.41–4.17, median of the differences -1.66 mg/L, 95% confidence interval [CI] -2.17 – -1.22 mg/L, p-value <0.01), corresponding to a median change from baseline of -40%. Interleukin-6 decreased from 2.51 ng/L (IQR 1.59–4.32 ng/L) to 2.22 ng/L (median of the differences -0.36 ng/L, 95%CI -0.70 – -0.01 ng/L, p-value 0.04), corresponding to a median change from baseline of -16%. No clinically relevant changes in lipid fractions were observed. Both leukocyte and thrombocyte count decreased (median change from baseline -7% and -4% respectively). Estimated glomerular filtration rate decreased with a mean change from baseline of -2%.

**Conclusion:**

In patients with chronic coronary artery disease and elevated hs-CRP, one-month exposure to colchicine 0.5 mg once daily was associated with a reduction of inflammatory markers. A small effect was seen on white blood cell count and platelet count, as well as a small decrease in estimated glomerular filtration rate.

## Introduction

Inflammation plays a pivotal role in the complex pathophysiology of atherothrombosis [1,2]. Part of the inflammatory response may be initiated by the crystallization of cholesterol in the lipid pool of the atheroma. Leukocyte chemotaxis and inflammatory response can compromise stability of thin capped fibroatheromata. In addition, the cholesterol crystals may pierce through the plaque cap causing direct disruption of the cap [3]. Important chemokines in these processes are interleukin (IL)– 1 and cytosolic multimeric protein complexes called inflammasomes [4–6].

Dampening the inflammatory response in atherosclerosis with anti-inflammatory drugs has yielded contradictory results. Anti-inflammatory treatment with canakinumab, a therapeutic monoclonal antibody targeting interleukin-1β, resulted in a detectable anti-inflammatory biochemical response and was associated with beneficial effects on major clinical outcomes in cardiovascular disease. Treatment with low-dose methotrexate however, did not show biochemical or clinical response [7,8]. Colchicine is an anti-inflammatory drug highly effective in reducing crystal induced inflammation in gout [9]. It is currently investigated as a potential anti-inflammatory drug in several atherosclerotic vascular disease states [10]. Although there is some evidence on its clinical efficacy in cardiovascular disease, the effects on downstream markers of inflammation may vary, and have not yet been investigated in patients with chronic coronary artery disease [11–14].

Colchicine has a narrow therapeutic index. First, competition with Cytochrome P450 3A4 (CYP3A4) or P-glycoprotein metabolizing drugs may lead to decreased clearance of colchicine or alter pharmacodynamics of CYP3A4 substrates such as statins. Second, colchicine in high dose can modulate myeloid cell lines due to its anti-proliferative properties [15,16]. Third, colchicine is relatively contra-indicated in patients with advanced renal insufficiency, although possible reno-protective properties of the drug are increasingly investigated in patients with renal disease [17].

The aim of this study was to investigate whether 30 days exposure to colchicine 0.5mg once daily leads to a reduction in inflammatory biomarkers in patients with chronic coronary artery disease and to investigate effects of treatment on lipid fractions, blood indices, and renal function.

## Methods

### Design and population

We conducted a prospective, open-label, clinical study with pre- and post-exposure blood testing. Accordingly, adult patients with chronic coronary artery disease were recruited from three Dutch hospitals. Recruitment took place from May 1^st^, 2017 to December 4^th^, 2018. Patients were eligible for participation if they had coronary artery disease proven by either invasive coronary angiography or computed tomography coronary angiography with an Agatston calcium score > = 400 units, a high sensitivity [hs]-CRP ≥2 mg/L and if they were considered clinically stable to the discretion of the caregiver. This cut-off value for hs-CRP was chosen to select patients with a pro-inflammatory status and to optimize comparability to prior clinical studies [7,18]. Exclusion criteria were an estimated glomerular filtration rate [eGFR] < 30 ml/min/1,73m^2^) or a serum creatinine > 150 μmol/L, the necessity to take colchicine for any other indication or concomitant drug use of strong CYP3A4 inhibiting drugs (verapamil, azithromycine, clarithromycin). Patients had to be treated for chronic coronary artery disease without active intercurrent illnesses, frailty or a limited life expectancy according to the discretion of their treating physician. Treatment with other anti-inflammatory drugs was permitted. Obesity was defined as a body mass index of 25 kg/m2 or more. Diabetes was defined by use of oral or parenteral glucose level modifying drugs. Statin intensity was categorized in accordance with the guidelines of the American College of Cardiology and American Heart Association, and high dose statin was defined as atorvastatin 40mg or 80mg dose equivalent [19].

### Exposure and outcome

Patients were supplied with colchicine 0.5mg once daily to be taken orally at the same time of the day. The pre-specified primary outcome of the present study was change of hs-CRP and IL-6 after 30 days of exposure to colchicine. Secondary outcomes were change in lipids, leukocyte count, thrombocyte count, red blood cell parameters, creatinine, and estimated renal function using the Chronic Kidney Disease–Epidemiology Collaboration (CKD-EPI) formula. Follow-up took place from May 1^st^, 2017 to February 1^st^, 2019.

### Laboratory assessment

Blood samples were taken before and after 30 days of exposure to colchicine. All samples were centrifuged (1500xg at 4°C for 15 minutes) and serum was kept stored at -80°C in separate containers. Levels of CRP and IL-6 were measured in a central core laboratory. Hs-CRP was measured using a research hs-CRP Elisa kit (Hycult Biotech #HK369, Uden, the Netherlands). The lower detection limit of this assay was 0.4ng/L and the inter- and intra-assay coefficients of variation were <6.9% and <6.3% respectively. IL-6 levels were measured by highly sensitive human IL-6 immunoassay (R&D Systems #D6050, Minneapolis, MN, USA) for which intra-assay and inter-assay coefficients of variation ranged from 4.2% to 6.4%. This assay had a sensitivity of 0.7 pg/mL.

The hematology parameters were determined with the XN9000 of Sysmex (Sysmex, Kobe, Japan). The serum LDL cholesterol was determined with the third-generation homogeneous enzymatic colorimetric assay, the serum creatinine with the second-generation enzymatic assay. These analyses were carried out in a routine setting under ISO15189 compliance.

### Statistical analysis

We estimated that using a sample size of 130 subjects would provide 80% power to detect a mean of the differences of -1 mg/dl in hs-CRP concentration, assuming a standard deviation of 4 mg/dl. Due to the expected non-normal distribution of differences, the minimal number of participants was extended with 15% to increase discriminative power in non-parametric testing [20].

Central tendencies and distribution of continuous parameters were displayed using mean or median with standard deviation or 25^th^ and 75^th^ percentile in case of normally and non-normally distributed variables respectively. Categorical variables were presented as proportions. Paired differences of parameters were evaluated and provided using the mean or median of the differences and the corresponding 95% confidence interval (CI). Standard errors and confidence intervals for medians in non-parametric distributions were computed using bias-corrected and accelerated bootstrapping. A Hodges–Lehmann estimator was used to provide a pseudo-median and confidence interval of the differences between non-parametric distributions. Formal hypothesis testing was done using a paired sample T–test for normally distributed differences and the Wilcoxon Signed Rank Test with continuity correction for non-normally distributed differences. The linear relationship of two continuous parameters was calculated using the Spearman's rank correlation coefficient.

For exploratory purposes, interaction of the estimated treatment effect in selected subgroups was tested using a mixed effects model with random intercepts and slopes with log transformation of outcome data. All subgroups were included in the final model to adjust for confounding.

All calculations were done with R (The R Foundation for Statistical Computing, version 3.6.0. using the packages “LMER” and “boot”).

### Ethical approval and funding

The study was approved by a central ethics committee (MEC-U, Nieuwegein, the Netherlands). All patients signed informed consent prior to participation. This work was supported by a governmental grant from The Netherlands Organisation for Health Research and Development [grant number 848015014]. The drug was supplied free of charge by TioPharma (Oud-Beijerland, the Netherlands). The funders and drug supplier had no role in study design, data collection and analysis, decision to publish, or preparation of the manuscript. The authors confirm that all ongoing and related trials for this drug/intervention are registered and the main trial is registered in the Australian Clinical Trials Registry (ACTRN12614000093684). There are no conflicts of interests by the authors.

## Results

**Fig 1:**
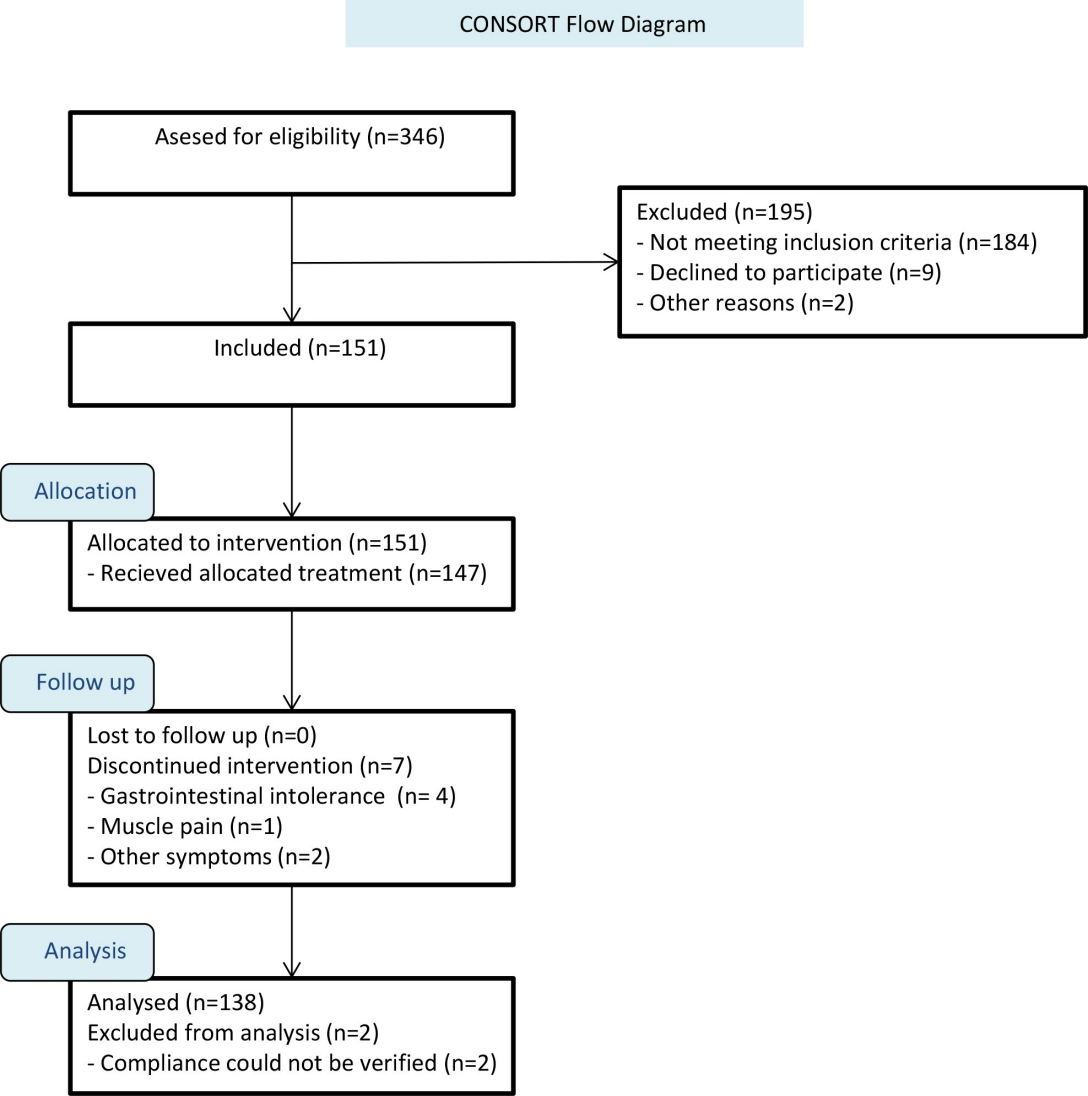
Flowchart of study. The figure shows selection of subjects and reason for exclusion. Abbreviation: hs-CRP: High sensitivity C-reactive Protein.

Blood samples were available in 337 patients. Major reasons for exclusion where hs-CRP <2 (n = 184) or intolerance to the drug (n = 7), mainly in the form of gastro-intestinal upset (Fig 1). 138 patients were included in the final analysis.

Median age was 65 years and 82% were male (Table 1). Of these, 115 (83%) had a previous acute coronary syndrome with the last event occurring a median of 23 months prior to inclusion (interquartile range [IQR] 9–76 months). Active smokers comprised 14% of the cohort and 20% had diabetes. All had single or dual antiplatelet therapy or anticoagulants, 88% were treated with statins and 59% with high dose statins.

**Table 1:** Baseline characteristics.

Demographics		n = 138
Age, years	Mean (SD)	65.1 (9.4)
Male		113 (81.9%)
Smoker		
Active smoker		19 (13.8%)
Former smoker		88 (63.8%)
BMI, kg/m2	Mean (SD)	28.7 (4.3)
**Cardiovascular medical history**		
Hypertension		80 (58.0%)
Diabetes		27 (19.6%)
Diabetes on insulin		11 (8.0%)
Prior acute coronary syndrome		115 (83.3%)
Time since last acute coronary syndrome, months	Median (IQR)	22.8 (9.3–75.8)
Prior percutaneous coronary stenting		124 (89.9%)
Left ventricular ejectionfraction, %	Mean (SD)	54.1 (9.9)
**Cardiovascular drug use**		
Dual antiplatelet therapy		57 (41.3%)
Any antiplatelet therapy or oral anticoagulants		138 (100.0%)
Betablocker or calciumchannelblocker		108 (78.3%)
ACE inhibitors or angiotensin receptor blockers		93 (67.4%)
Any of the above mentioned anti-hypertensives		125 (90.58%)
Statin		121 (87.7%)
High dose statin		71 (58.7%)
Any lipid lowering drug:		132 (95.7%)

Statin intensity was categorized in accordance with the guidelines of the American College of Cardiology and American Heart Association, and high dose statin was defined as a atorvastatin 40mg or 80mg dose equivalent [19]. Abbreviations: ACE, angiotensin converting enzyme; BMI, body mass index; IQR, inter quartile range; kg, kilogram; SD, standard deviation.

### Inflammatory biomarkers

**Fig 2:**
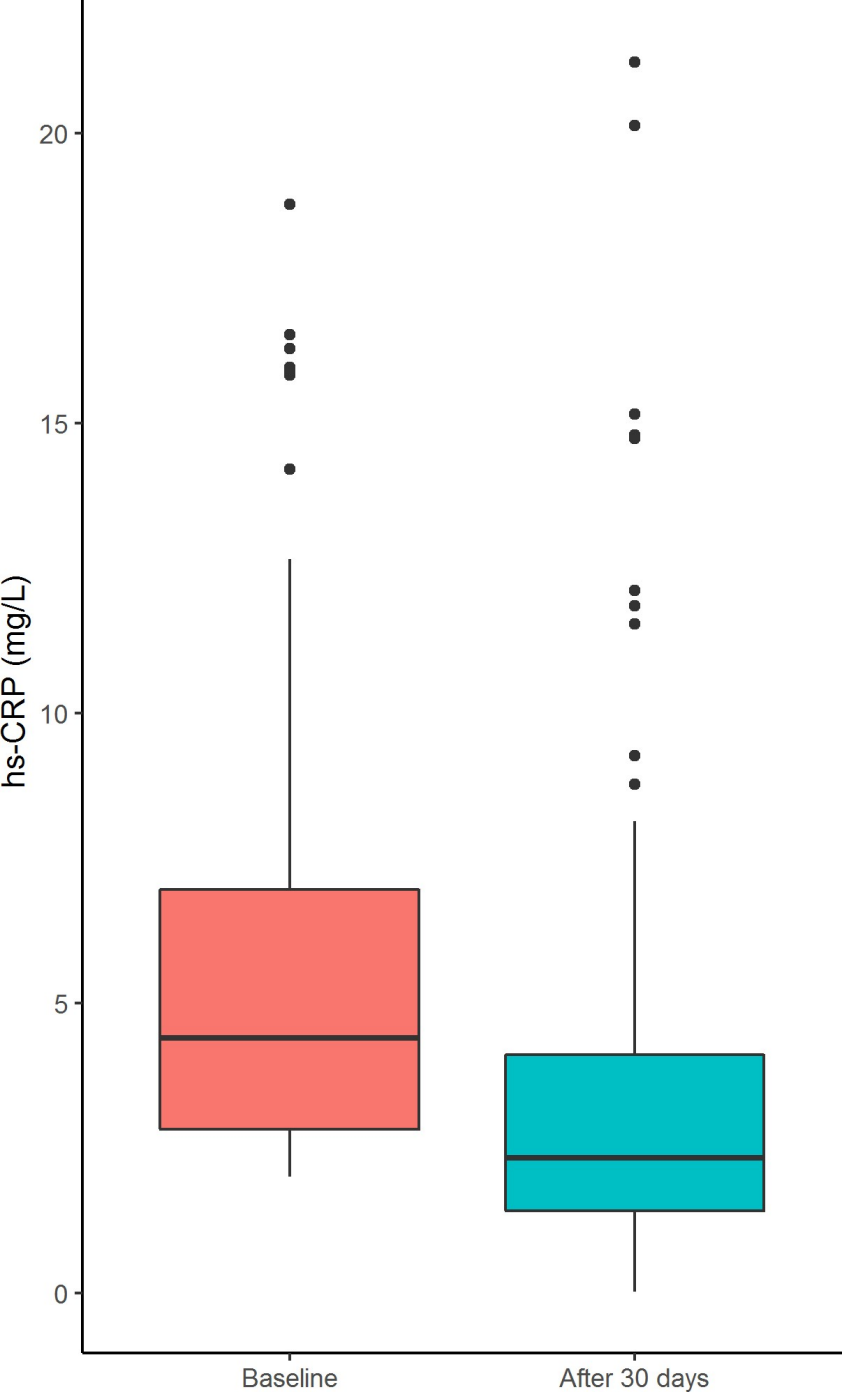
Boxplot representing change of hs-CRP levels after 30 days of colchicine exposure. The figure shows the change in median hs-CRP levels after 30 days of colchicine exposure. Abbreviation: hs-CRP: High sensitivity C-reactive Protein.

**Fig 3:**
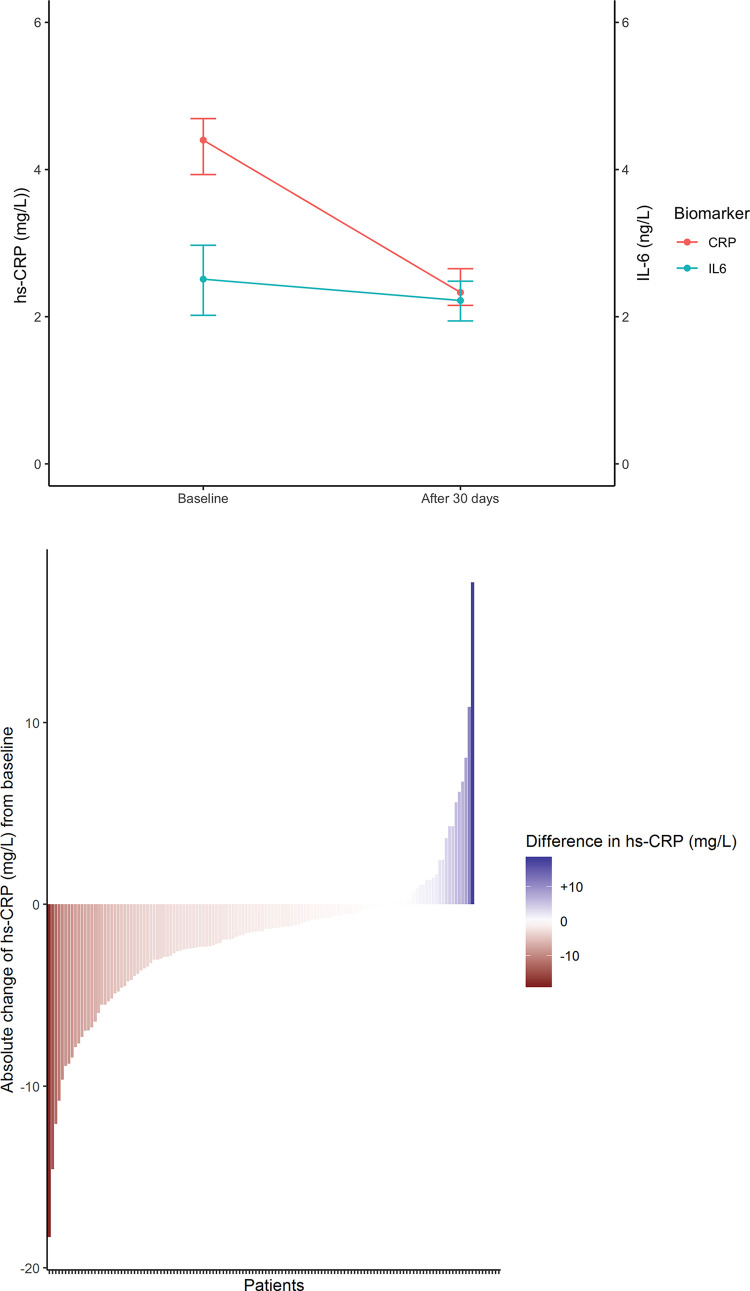
**a**. Median change in hs-CRP. The figure shows the median change in hs-CRP and IL-6 levels after 30 days of colchicine exposure. Horizontal lines represent 95% confidence intervals. Abbreviations: hs-CRP, High sensitivity C-reactive Protein; IL-6, interleukin-6. **b**. Absolute change in hs-CRP & IL-6. The figure shows a waterfall plot of change in hs-CRP from baseline to 30 days after colchicine exposure. Vertical bars in the plot represent individual patients. Abbreviations: hs-CRP, high sensitivity C-Reactive Protein; IL-6, interleukin-6.

Median baseline hs-CRP was 4.40 mg/L (IQR 2.83–6.99 mg/L). After one month hs-CRP was 2.33 mg/L (IQR 1.41–4.17, median of the differences -1.66 mg/L, 95%CI -2.17 –-1.22 mg/L, p-value <0.01, Fig 2) corresponding to a median change from baseline of -40% (Table 2, Fig 3A). In 81% of patients hs-CRP decreased, with 36% of patients reaching a hs-CRP <2 (Fig 3B).

**Table 2:** Inflammatory markers and lipids.

			**Visit 1**	**Visit 2**	**Mean or median of differences**	**95% CI**	**P-value**	**% change from baseline**
**Inflammatory Markers**								
Interleukin-6	ng/L	median	2.51	2.22	-0.36	(-0.70 to -0.01)	0.04	-16%
hs-CRP	mg/L	median	4.40	2.33	-1.66	(-2.17 to -1.22)	<0.01	-40%
**Lipids**			**Visit 1**	**Visit 2**	**Mean or median of differences**	**95% CI**		**% change from baseline**
Total Cholesterol	mmol/l	Mean	4.07	3.96	-0.07	(-0.16 to 0.01)	0.091	NS
HDL Cholesterol	mmol/l	Mean	1.16	1.13	-0.03	(-0.05 to -0.01)	<0.01	-3%
LDL cholesterol	mmol/l	Mean	2.38	2.24	-0.07	(-0.13 to 0.00)	0.04	-2%
Triglycerides	mmol/l	Mean	2.04	2.13	0.08	(-0.05 to 0.21)	0.21	NS

Abbreviations: CI, confidence interval; HDL, high density lipid; hs, high sensitivity; LDL, low density lipid; NS, not significant.

Median baseline IL-6 was 2.51 ng/L (IQR 1.59–4.32 ng/L) and after one month 2.22 ng/L (median of the differences -0.36 ng/L, 95%CI -0.70 –-0.01 ng/L, p-value 0.01), corresponding to a median change from baseline of -16% (Fig 3A).

**Fig 4:**
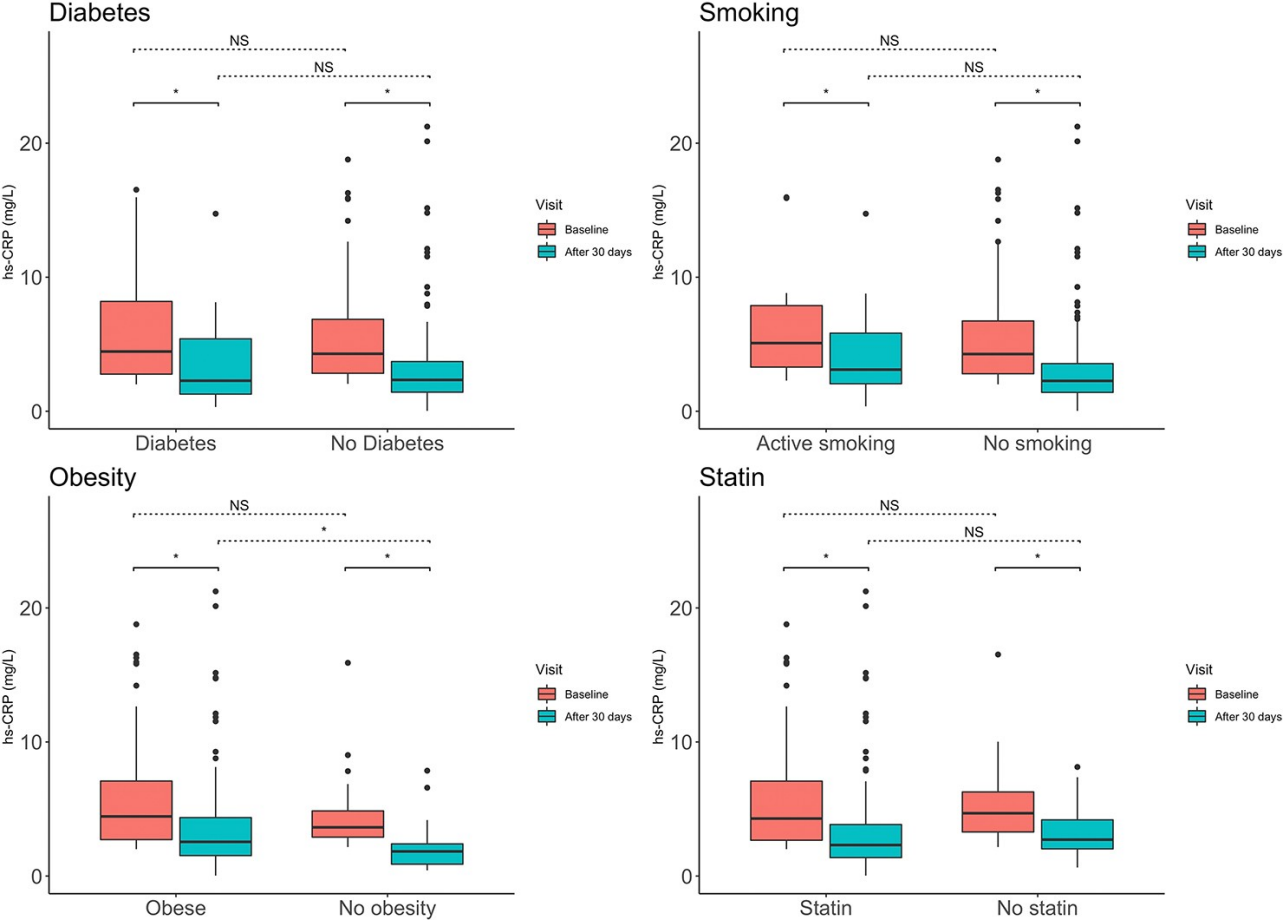
Subgroup changes in hs-CRP. The figure shows the median change in hs-CRP after 30 days of colchicine exposure in four subgroups. An asterisk depicts p-values < 0.05 for the paired and non-paired comparisons. In all subgroups hs-CRP decreased significantly. There was no significant interaction of the subgroups and change in hs-CRP. Abbreviations: hs-CRP, high sensitivity C-Reactive Protein; NS, not significant.

In 57% of patients hs-CRP decrease showed concordance with IL-6 decrease. Overall, a change in hs-CRP showed a moderate correlation with change in IL-6 (R = 0.41, p <0.001). The median decrease of hs-CRP and IL6 was consistent among various pro-inflammatory phenotypes such as diabetes, smoking, obesity and independent of statin use. There were no significant differences in baseline level of hs-CRP or IL-6 between these groups. (Table 3 and Fig 4).

**Table 3:** Differences in inflammatory markers between subgroups.

	Visit 1 hs-CRP [IQR]	Visit 2 hs-CRP [IQR]	P-value for interaction	Visit 1 IL6 [IQR]	Visit 2 IL6 [IQR]	P-value for interaction
**Clinical subgroup**						
Diabetes	4.46 [2.56–8.42]	2.28 [1.23–5.53]	0.329	2.96 [1.97–4.72]	3.09 [1.75–3.67]	0.743
No diabetes	4.29 [2.83–6.86]	2.34 [1.42–3.77]		2.40 [1.49–4.10]	2.13 [1.38–3.39]	
P-value for within differences	0.723	0.896		0.147	0.115	
Active smoking	5.08 [2.83–8.33]	3.10 [2.05–5.83]	0.893	2.45 [1.50–3.74]	2.48 [1.90–4.62]	0.084
No active smoking	4.27 [2.76–6.86]	2.27 [1.41–3.56]		2.62 [1.59–4.56]	2.17 [1.37–3.25]	
P-value for within differences	0.256	0.087		0.753	0.160	
Obesity	4.45 [2.67–7.10]	2.55 [1.52–4.48]	0.156	2.70 [1.76–4.53]	2.30 [1.45–3.58]	0.063
No obesity	3.63 [2.86–4.99]	1.84 [0.69–2.46]		1.76 [0.97–3.44]	1.76 [0.82–3.01]	
P-value for within differences	0.304	0.010		0.060	0.173	
Statin	4.29 [2.67–7.08]	2.31 [1.38–3.91]	0.097	2.64 [1.55–4.53]	2.26 [1.45–3.58]	0.483
No statin	4.69 [3.29–6.27]	2.72 [2.02–4.21]		1.97 [1.64–3.44]	1.77 [0.57–2.69]	
P-value for within differences	0.551	0.274		0.403	0.145	

Abbreviations: hs-CRP, high sensitive C Reactive Protein; IL, interleukin; IQR, interquartile range.

### Lipids

At baseline mean LDL cholesterol level was 2.38 mmol/L. No significant changes were observed in levels of LDL cholesterol, total cholesterol and triglycerides (Table 2). HDL cholesterol decreased from a mean of 1.16 mmol/L to 1.13 mmol/L (median of the differences -0.03 mmol/L, 95% CI -0.05 to -0.01 mmol/L, p-value <0.01), corresponding with a median change from baseline of -3%.

### Hematologic markers

No change was seen in concentration of hemoglobin, hematocrit or erythrocytes (Table 4). The mean corpuscular volume of the erythrocytes went from baseline 91.47 to 91.17 femtoliter (mean of the differences -0.35 femtoliter, 95%CI -0.68 to -0.03, p-value 0.03).

**Table 4:** Haematology and renal function.

			**Visit 1**	**Visit 2**	**Mean or median of differences**	**95% CI**	**P-value**	**% change from baseline**
**Haematology**								
Hemoglobine	(mmol/L)	Mean	9.08	9.09	0.02	(-0.05 to 0.09)	0.59	NS
Hematocrit	L/L	Mean	0.44	0.44	0.00	(-0.01 to 0.00)	0.28	NS
Erytrocytes	(x 10^12/L)	Mean	4.87	4.87	0.00	(-0.04 to 0.04)	0.93	NS
MCV	(fl)	Mean	91.47	91.17	-0.35	(-0.68 to -0.03)	0.03	0%
Leukocytes	(x 10^9/L)	Median	6.99	6.62	-0.47	(-0.74 to -0.19)	<0.01	-7%
Trombocytes	(x 10^9/L)	Median	237.50	231.00	-11.50	(-16.50 to -6.50)	<0.01	-4%
**Renal function**			**Visit 1**	**Visit 2**	**Mean or median of differences**	**95% CI**	**P-value**	**% change from baseline**
eGFR	(ml/min/1,73m2)	Mean	76.81	74.99	-1.94	(-3.46 to -0.42)	0.01	-2%
Creatinine	(μmol/L)	Mean	87.90	89.53	1.69	(0.10 to 3.28)	0.04	1%

Abbreviations: CI, confidence interval; eGFR, estimated glomerular filtration rate; MCV, mean corpuscular volume; NS, not significant.

The number of leukocytes decreased from a median of 6.99 to 6.62 x 10^9/l (mean of the differences -0.47 x 10^9/l, 95% CI –0.47 to -0.74, p-value <0.01), corresponding to a median change from baseline of -7%. The number of thrombocytes decreased from a median of 237.50 to 231.00 x 10^9/l (median of the differences -11.50 x 10^9/l, 95%CI -16.50 to -6.50, p-value <0.01), corresponding to a median change from baseline of -4%. Leukocyte changes were significantly correlated with changes in hs-CRP (R = 0.23, p < 0.001).

### Renal function

Creatinine increased from a mean of 87.90 to 89.53 μmol/L (mean of the differences 1.69 μmol/L, 95%CI 0.10 to 3.28 μmol/L, p-value 0.01), corresponding to a mean change from baseline of 1% (Table 3). Derived mean eGFR was 76.81 ml/min/1.73m^2^ at baseline and decreased to 74.99 ml/min/1.73m^2^ after 30 days (mean of the differences -1.94 ml/min/1.73m^2^, 95%CI -3.28 to -0.42, p-value 0.01), corresponding to a mean change from baseline of -2%. Mild or moderate renal impairment (eGFR 30 to 60 ml/min/1.73m^2^) was seen in 11% of patients at baseline and 14% of patients at follow-up. Change in eGFR did not correlate significantly with the change in hs-CRP (R 0.07, p = 0.435).

## Discussion

This study showed that patients with chronic coronary artery disease and a pro-inflammatory state defined by hs-CRP ≥ 2 mg/L, had a significant decrease in both hs-CRP and IL-6 after one month of colchicine exposure. No clinically relevant changes in lipid spectrum were observed. A small decrease in mean corpuscular volume was observed, as well as a decrease in leukocyte and thrombocyte number and a small decrease in estimated glomerular filtration rate. The present study extends our knowledge of the anti-inflammatory effect of 0.5mg colchicine once daily to patients with chronic coronary artery disease.

### Anti-inflammatory effects

The direction and extent of the change in inflammatory markers after exposure to colchicine in this pro-inflammatory population with coronary artery disease compares to observations from previous studies in heart failure and patients with metabolic syndrome. Although absolute reduction of hs-CRP and IL-6 were larger in these studies in which baseline levels were higher, the relative reduction was similar. The reduction in the current study was achieved with a lower dose of colchicine [21,22]. Systemic hs-CRP and IL-6 reduction have not been observed when administrating colchicine directly after acute myocardial infarction [14,23,24]. This may represent limitations in the anti-inflammatory effect of the drug in highly inflammatory conditions such a reperfusion injury and extended necrosis following acute myocardial infarction [25,26].

The magnitude of hs-CRP and IL-6 reduction found in the current study is similar to the effect of canakinumab used in the Canakinumab Antiinflammatory Thrombosis Outcome Study (CANTOS) [7]. Although hs-CRP <2 mg/L is associated with a lower risk for future cardiovascular events, hs-CRP itself carries no causal relationship to such incidents [18]. Inference to the magnitude of any clinical effect based on the current observations is thus explorative. Other surrogate markers for clinical outcomes are suggested to have a directly proportional relationship with hs-CRP concentration. For example, a change in hs-CRP has been shown to be associated in a linear manner with low attenuation plaque volume, a radiologic marker of plaque stability [27].

In contrast to hs-CRP, the evidence for a causal relation to levels of IL-6 and in particular IL– 1 beta and atherothrombosis has become more rigid after observational Mendelian randomization studies and results of the CANTOS trial [7,28–30]. A causative role for these two cytokines in disease progression is further implied by the absence of clinical benefit using the anti-inflammatory drug methotrexate, which did not yield a change in hs-CRP or IL-6 levels [8].

IL– 1 beta is an upstream biomarker of IL-6 and there are data suggesting that high doses of colchicine can reduce IL– 1 beta release. However, such an effect has not been described in patients with chronic coronary artery disease [31]. The absence of a clinical effect in the low dose canakinumab arm with the smallest change in inflammatory biomarkers may suggest a certain biomarker threshold to reach clinical effect. This hypothesis is further substantiated by the absence of clinical effect in patients not reaching below-median levels of IL-6 in the CANTOS trial [32].

### Blood count effects

A small reduction in leukocyte and thrombocyte count was observed during one month of colchicine exposure. This finding is not unexpected. Colchicine affects leukocyte adhesion and chemotaxis and also has an anti-proliferative effect on leukocytes, mediated by the ability to disrupt the cytoskeleton. Colchicine irreversibly binds with tubulin to form an intracellular tubulin complex preventing the formation of microtubules [33,34]. In high doses the drug will arrest mitosis in metaphase as it precludes chromosome separation [15]. Data in acute myocardial infarction, gout or Familial Mediterranean Fever do not support the possibility that these changes translate into clinically relevant adverse effect such as increased likelihood of bleeding [14,35]. In patients with recent myocardial infarction, no numerical differences in total white blood count or lymphocytes between active and placebo drugs were seen twelve months after treatment initiation, and similar rates of infection in general were seen, but higher incidences of pneumonia (0.9% versus 0.4% in patients on placebo (p = 0.03) [14]. Treatment with canakinumab resulted in a small increase in the incidence of fatal infection, albeit with a low absolute risk (0.31 versus 0.18 events per 100 person years) [7].

### Renal effects

A small increase in serum creatinine and a corresponding decrease of eGFR was observed. Excretion of colchicine takes place mainly via the enterohepatic circulation (80%) and in part via renal excretion (20%) [9,36]. Whether current observations are a direct drug effect on glomerular filtration should be investigated further. Future analysis should include to what extent renal function is affected during long-term administration. The absence of correlation between change in eGFR and magnitude of hs-CRP reduction suggests that the effect of colchicine on eGFR is different from the anti-inflammatory effects of colchicine and may be due to a direct hemodynamic effect on the glomerulus [37,38]. Another effect could be the Hawthorne effect, i.e. an indirect effect of trial participation. Whether the patients increased their compliance with drugs affecting glomerular hemodynamics such as angiotensin converting enzyme inhibitors was not be assessed in the current study setup, but is a known effect in trial participants [39,40].

### Limitations

A methodological limitation of our study is the paired testing with the absence of a parallel control group. This is inherent to the design of the study as it was part of the run-in phase prior to randomization of three sites in a clinical trial. Due to the selection of hs-CRP ≥2, part of the change in hs-CRP may be explained by regression to the mean or natural course. However, changes in hs-CRP are more pronounced than those observed in the placebo arm of the similar CANTOS population (change of -40% in this cohort versus -17% in CANTOS), suggesting the CRP decrease is perhaps only partially influenced by preselection and natural course. In addition, no change in IL-6 was seen in the placebo arm of CANTOS [7].

Finally, the subgroup analyses have an exploratory purpose only. Since the study was not designed to assess differences between subgroups, the limited sample size increases risk for type 2 errors in these observations.

## Conclusion

One-month exposure to low-dose colchicine was associated with a reduction of the inflammatory markers hs-CRP and IL-6 in patients with chronic coronary artery disease and baseline hs-CRP ≥2 mg/L. An effect was seen on estimated glomerular filtration rate, leukocyte and thrombocyte cell count, which warrants placebo-controlled and longer follow-up of these parameters. Whether the anti-inflammatory and other effects observed in this study are solely contributable to colchicine should be confirmed in placebo-controlled assessment. Whether this effect translates into a clinical benefit has to be awaited. Observations thus far support ongoing clinical research in colchicine as anti-inflammatory drug in atherosclerosis.

## Supporting information

S1 Checklist TREND checklist. (DOC)

S1 File Substudy protocol. (DOCX)
