# Supplementary material for: Short-term effect of low-dose colchicine on inflammatory biomarkers, lipids, blood count and renal function in chronic coronary artery disease and elevated high-sensitivity C-reactive protein
Source: PLoS One. 2020 Aug 31;15(8):e0237665. doi: 10.1371/journal.pone.0237665 (PMC7458326; doi:10.1371/journal.pone.0237665)
Supplement: S1 File — (DOCX) [file pone.0237665.s002.docx]

# SUBSTUDY PROTOCOL

**Main study : Low-dose colchicine for secondary prevention of cardiovascular disease – LoDoCo2 trial**

**Substudy: Run-in laboratory substudy**

Main protocol

EudraCT number: 2015-005568-40

Australian Clinical Trials Registry: ACTRN12614000093684

Protocoldate 9 January 2017

Principal investigators:

Jan Hein Cornel

Arend Mosterd

Dominique de Kleijn

Willem Bax

Aernoud Fiolet

Tjerk Opstal

Max Silvis

**This Protocol contains confidential information.**

**Do not share this with other parties not bound by a confidentiality agreement.**

# PROTOCOL SYNOPSIS

| Title | Substudy on laboratory results during the run-in of the Low-Dose Colchicine for secondary prevention of cardiovascular disease |
| --- | --- |
| Principal Investigators | Jan Hein Cornel  Arend Mosterd  Dominique de Kleijn  Willem Bax  Aernoud Fiolet  Tjerk Opstal  Max Silvis |
| Study hypothesis | Low-dose colchicine in patients with established stable coronary artery disease will decrease inflammatory biomarkers |
| Study Design | Prospective, multicentre, open label, study of colchicine 0.5mg once daily during 30 days. |
| Population and recruitment of participants | Patients with stable coronary artery disease from cardiology practices in The Netherlands. |
| Primary Objective | The primary objective of this study is to evaluate changes in inflammatory biomarkers during treatment with colchicine 0.5mg once daily |
| Secondary Objectives | The secondary objectives of this study are to evaluate changes on bloodcounts, renal function and lipids during treatment with colchicine 0.5mg once in patients with stable coronary artery disease. |
| Inclusion Criteria | 1. Age >35 and <82 years 2. Proven coronary artery disease; as evidenced by coronary angiography, CT coronary angiography or a Coronary Artery Calcium Score (Agatston score >400). Individuals with a history of bypass surgery are only eligible if they have undergone coronary artery bypass surgery more than 10 years before, or have angiographic evidence of graft failure or have undergone percutaneous intervention since their bypass surgery 3. Clinically stable for at least six months |
| Exclusion criteria | 1. Women who are pregnant, breast feeding or may be considering pregnancy during the study period 2. Renal impairment as evidenced by a serum creatinine >150 µmol/l or estimated glomerular filtration rate (eGFR) <50mL/min/1.73m2 3. Severe heart failure – systolic or diastolic New York Heart Association Functional classification 3 or 4 4. Moderate or severe valvular heart disease considered likely to require intervention 5. Dependency or frailty or an estimated life expectancy < 5 years 6. Peripheral neuritis, myositis or marked myo-sensitivity to statins 7. Requirement for long term colchicine therapy for any other reason 8. Current enrollment in another trial |
| Treatment Regimen | Colchicine 0.5mg once daily during 30 days. |

# TABLE OF CONTENTS

[PROTOCOL 1](#_Toc29746745)

[SUBSTUDY LoDoCo2 1](#_Toc29746746)

[PROTOCOL SYNOPSIS 2](#_Toc29746747)

[TABLE OF CONTENTS 4](#_Toc29746748)

[LIST OF ABBREVIATIONS AND RELEVANT DEFINITIONS 5](#_Toc29746749)

[1. INTRODUCTION AND RATIONALE 5](#_Toc29746750)

[1.1 Inflammation in atherosclerosis 5](#_Toc29746751)

[2. OBJECTIVES 6](#_Toc29746752)

[3. STUDY DESIGN 6](#_Toc29746753)

[4. STUDY POPULATION 6](#_Toc29746754)

[4.1 Population 6](#_Toc29746755)

[4.2 Inclusion criteria 6](#_Toc29746756)

[4.3 Exclusion criteria 6](#_Toc29746757)

[4.4 Sample size 7](#_Toc29746758)

[4.5 Study flow diagram 7](#_Toc29746759)

[5. TREATMENT OF SUBJECTS 7](#_Toc29746760)

[5.1 Investigational product/treatment 7](#_Toc29746761)

[5.2 Additional study treatment 7](#_Toc29746762)

[6. INVESTIGATIONAL PRODUCT 7](#_Toc29746763)

[6.1. Safety monitoring 8](#_Toc29746764)

[7. METHODS 8](#_Toc29746765)

[7.1 Study parameters 8](#_Toc29746766)

[7.2Study procedures 8](#_Toc29746767)

[7.4 Schedule of assessment 10](#_Toc29746768)

[7.5 Withdrawal of individual subjects 10](#_Toc29746769)

[8. SAFETY 11](#_Toc29746770)

[8.1 Safety monitoring 11](#_Toc29746771)

[8.2 Site monitoring 11](#_Toc29746772)

[8.3 (Serious) Adverse events and suspected, unexpected severe adverse reactions. 11](#_Toc29746773)

[9. STATISTICAL ANALYSIS 12](#_Toc29746774)

[10. ETHICAL CONSIDERATIONS 12](#_Toc29746775)

[10.1 Regulation statement 12](#_Toc29746776)

[10.2 Recruitment and consent 13](#_Toc29746777)

[11. ADMINISTRATIVE ASPECTS, MONITORING AND PUBLICATION 13](#_Toc29746778)

[11.1 Handling and storage of data and documents 13](#_Toc29746779)

[11.2 Amendments 13](#_Toc29746780)

[12. RISK ANALYSIS 13](#_Toc29746781)

[14. REFERENCES 13](#_Toc29746782)

# LIST OF ABBREVIATIONS AND RELEVANT DEFINITIONS

| **CT** | **Computed tomography** |
| --- | --- |
| **(hs-)CRP** | **(high sensitive-) C-Reactive protein** |
| **(e)GFR** | **(estimated) Glomerular filtration rate** |
| **HR** | **Hazard ratio** |
| **ICH-GCP** | **International Conference on Harmonisation of Technical Requirements for Registration of Pharmaceuticals for Human Use (ICH) of Good Clinical Practice (ICH-GCP)** |
| **LoDoCo** | **Low-dose Colchicine for Secondary Prevention of Cardiovascular Disease** |
| **NLRP3** | **NACHT, LRR and PYD domains-containing protein 3** |
| **(S)AE** | **(Serious) adverse event** |
| **SUSAR** | **Suspected unexpected serious adverse reactions** |

# 1. INTRODUCTION AND RATIONALE

### 1.1 Inflammation in atherosclerosis

Despite major advances in treatment, the burden of cardiovascular disease remains high. Worldwide, an estimated 17.5 million people died from cardiovascular diseases in 2012, of which an estimated 7.4 million due to coronary artery disease.^1^ It is the second most common cause of mortality in the Netherlands with almost 40,000 deaths in 2014. A quarter of these is directly related to coronary artery disease. ^2^

The recognition that atherosclerosis is an inflammatory process has changed attitudes to the management of coronary heart disease and introduced into cardiology the concept of plaque instability.^3^ The histo-pathologic features of unstable plaques are now well recognized as a thin fibrous cap, a large lipid pool and an inflammatory infiltrate.^4^ Changes within the plaque are characterized by the inflammatory infiltrate and release of proteolytic enzymes that can lead to erosion or rupture of the plaque surface, exposing atherosclerotic material to the blood and initiating coronary thrombosis.^5^ Despite recognition of the central role of plaque instability in the pathogenesis of unstable coronary syndromes, a lack of understanding of the underlying inflammatory mechanisms leading to plaque instability has hampered efforts for the development of new treatments to prevent plaque rupture and related complications.

Previous attempts to modulate inflammation within atherosclerotic plaque have been unsuccessful. The search for specific potential modulators of the inflammatory process which leads to plaque instability has included many possible targets, but few have the promise needed to enter a large scale clinical trial. ^6^

Colchicine is an anti-inflammatory drug highly effective in reducing crystal induced inflammation in gout.^7^ It is currently investigated as a potential anti-inflammatory drug in several atherosclerotic vascular disease states. Although there is some evidence on its clinical efficacy in cardiovascular disease, the effects on downstream markers of inflammation may vary, and have not yet been investigated in patients with chronic coronary artery disease. ^8–10^

Colchicine has a narrow therapeutic index. First, competition with Cytochrome P450 3A4 (CYP3A4) or P-glycoprotein metabolizing drugs might lead to decreased clearance of colchicine or alter pharmacodynamics of CYP3A4 substrates such as statins. Second, colchicine in high dose can modulate myeloid cell lines due its anti-proliferative properties. ^11,12^ Third, colchicine is relatively contra-indicated in patients with advanced renal insufficiency, although possible reno-protective properties of the drug are increasingly investigated in patients with renal disease. ^13^

# 2. OBJECTIVES

The aim of this study is to investigate whether colchicine 0.5mg once daily leads to a reduction in inflammatory biomarkers in patients with chronic coronary artery disease and to investigate effects of treatment on lipid fractions, blood indices, and renal function.

# 3. STUDY DESIGN

This study is a substudy in patients considered for participation in a larger clinical trial (the LoDoCo2 trial). This substudy uses a prospective, multicentre, open label design.

# 4. STUDY POPULATION

### 4.1 Population

The study population will consist of male/female patients aged 35 – 82 years with stable coronary artery disease (proven by coronary angiography, computed tomography (CT) coronary angiography or a Coronary Artery Calcium Score > 400 Agatston Units).

Participants will be recruited from The Netherlands. The recruiting centres will be cardiology practices from three large general hospitals.

### 4.2 Inclusion criteria

1. Age >35 and <82 years
2. Proven coronary artery disease; as evidenced by coronary angiography, CT coronary angiography or a Coronary Artery Calcium Score (Agatston score >400). Individuals with a history of bypass surgery are only eligible if they have undergone coronary artery bypass surgery more than 10 years before, or have angiographic evidence of graft failure or have undergone percutaneous intervention since their bypass surgery
3. Clinically stable for at least six months

### 4.3 Exclusion criteria

1. Women who are pregnant, breast feeding or may be considering pregnancy during the study period
2. Renal impairment as evidenced by a serum creatinine >150 µmol/l or estimated glomerular filtration rate (eGFR) <50mL/min/1.73m2
3. Severe heart failure – systolic or diastolic New York Heart Association Functional classification 3 or 4
4. Moderate or severe valvular heart disease considered likely to require intervention,
5. Dependency, frailty or a predicted life expectancy < 5 years
6. Peripheral neuritis, myositis or marked myo-sensitivity to statins
7. Requirement for long term colchicine therapy for any other reason
8. Current enrollment in another trial

### 4.4 Sample size

It is estimated that using a sample size of 130 subjects will provide 80% power to detect a mean of the differences of -1 mg/dl in hs-CRP concentration, assuming a standard deviation of 4 mg/dl. Due to the expected non-normal distribution of differences, the minimal number of participants will then be extended by 15% to increase discriminative power in non-parametric testing. ^14^

### 4.5 Study flow diagram


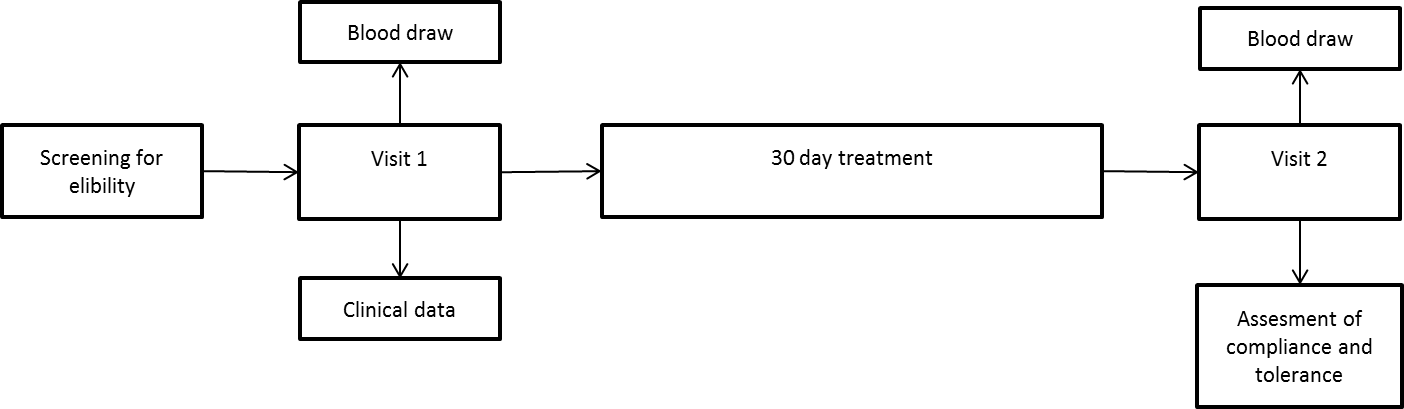


#

# 5. TREATMENT OF SUBJECTS

### 5.1 Investigational product/treatment

The investigational product is colchicine (0.5mg) prescribed once daily. The drugs will be supplied by Aspen Teva Pharmaceuticals in the Netherlands (produced by Tiofarma).

### 5.2 Additional study treatment

No additional treatment will be given for the purpose of this trial, apart from standard usual care for those with coronary artery disease. This may consist but is not limited to mono or dual therapy with thrombocyte aggregation inhibitors, lipid lowering drugs, angiotensin-converting enzyme – inhibitors, beta blockers and/ or calcium channel blockers.

# 6. INVESTIGATIONAL PRODUCT

Teva Pharmaceuticals has the market authorisation for colchicine PCH. The manufacturer of colchicine PCH is Tiofarma. Tiofarma will produce and supply colchicine PCH and matching placebo to the central pharmacy.

### Safety monitoring

#### Precautionary measures

Colchicine 0.5mg once daily is the reduced dosage recommended by the 2012 American College of Rheumatology Guidelines for Management of Gout and the FDA prescription information for Colcrys in patients with impaired renal function and for patients with concomitant use of CYP3A4 or P-glycoprotein inhibitors. ^15,16^

#### Concomitant drugs

Patient using the following drugs will not be able to participate

- Macrolide antibiotics: clarithromycin (similar to the Australian protocol), erythromycin, azitromycin
- Antimycotics (tiazole and imidazolederivates): ketoconazole, traconazole and voriconazole
- Protease inhibitors & ant-retroviral drugs: ritonavir, lopinavir, tipranavir, atazanavir, darunavir, indinavir, saquinavir and cobicistat
- Anti-arrhythmic drugs: verapamil
- Immunosuppressant: cyclosporine

# 7. METHODS

### 7.1 Study parameters

Demographical and clinical parameters for patients will be collected. The primary object are inflammatory markers and regular blood chemistry.

### Study procedures

#### 7.2.1. Screening and enrolment.

Potential participants will be screened by cardiologists, physicians or research professionals in out-patient clinics from cardiology practices from routine scheduled clinical visits and from post-myocardial infarction rehabilitation programs. Patients will be introduced to the study and provided information. After signing the informed consent they will be supplied open label colchicine 0.5mg once daily for the duration of 30 days.

#### 7.2.2 Baseline data assessment

Baseline and follow-up data will be registered on an (electronic) case report form. After signature of informed consent and before the start of the open label run-in phase baseline demographical data, medical and cardiovascular history, cardiovascular risk factors, and concomitant drug use will be collected.

##### 7.2.2.1 Clinical data

Data that will be collected include, but are not limited to:

- Demographical/ risk factors
  - Age, sex, ethnicity (Caucasian or non-Caucasian), smoking status (current/ never or discontinued >2 years ago/ discontinued <2 years ago), weight, length, family history of cardiovascular disease.
- Cardiovascular history
  - Diabetes mellitus, hypertension
  - Extent of coronary artery disease (single vessel, multivessel), date of last event, revascularisation (surgical or percutaneous), prior myocardial infarction
  - Peripheral artery disease, with or without intervention (percutaneous, bypassing, amputation), prior atherosclerotic stroke will be captured per vascular bed
  - Atrial tachyarrhythmia’s (atrial fibrillation/flutter)
- Concomitant drug use
  - Cardiovascular:
    - Antiplatelet therapy
    - Anticoagulation, by vitamin K antagonist or direct oral anticoagulants
    - Beta-blockers, calcium channel blockers
    - Angiotensin-converting enzyme – inhibitors or angiotensin receptor blockers
    - Lipid lowering drugs (statins, cholesterol uptake inhibitors, proprotein convertase subtilisin/kexin type 9 [PCSK9] inhibitors)
    - Diuretics
    - Other drugs
  - Other
    - Allopurinol

##### 7.2.2.2. Laboratory data prior to participation

Renal function prior to study participation is assessed based on measurement of serum creatinine and calculated creatinine clearance expressed as estimated glomerular filtration rate, (eGFR) using the method available in the local laboratory (i.e. Cockcroft-Gault, Modification of Diet in Renal Disease [MDRD], Chronic Kidney Disease Epidemiology Collaboration [CKD-EPI]). Available renal function at run-in should not be older than six months.

##### 7.2.2.3 Laboratory data prior to be collected

|  | **Biomarker** | **Time point of**  **assessment** |
| --- | --- | --- |
| Haematology | - Hemoglobin - Hematocrit - Mean corpuscular volume - Thrombocytes - Leukocytes | V1, V2 |
| Renal function | - Serum creatinine - Estimated glomerular filtration rate | V1, V2 |
| Lipidology | - Total Cholesterol - HDL - Triglycerides - LDL | V1, V2 |
| Glycemic status | - HbA1C | V1, V2 |
| Inflammation | - hsCRP - IL6 | V1, V2 |

#### 7.3.1 Follow-up & data assessment

Patients will return to the research nurse after one month. At this moment new blood will be drawn and their compliance to the drug as well as intolerance will be assessed. This point form the end of this study.

Patients and their general practitioners will be encouraged to report any occurring cardiac problems, hospitalisations or adverse reactions in between visits to coordinating research professionals or their cardiologist.

### 7.4 Schedule of assessment

At the first visit, once consent has been given, participants will begin their 30 day open label run-in trial of colchicine.

| **Visit** | **V1** | **V2*** |
| --- | --- | --- |
| **Week** | **0** | **4** |
| Visit window (days) |  | -7/+7 |
| Obtain informed consent | x |  |
| In- and exclusion criteria | x | x |
| Relevant medical and  cardiovascular history | x |  |
| Renal function** | x |  |
| Available laboratory data*** | x | x |
| Concomitant medication | x | x |
| Open label colchicine | x |  |
| Blood sampling | x | x |

* Visit 2 and planned assessments take place irrespectively of colchicine tolerance
** Renal function should not be older than six months at the moment of run-in
*** Assessed for routine out-patient clinical follow-up purposes or during hospitalization.

### 7.5 Withdrawal of individual subjects

Participants may withdraw from the study at any time. For the purpose of this study only patients compliant to the study medication will be analysed.

Participants who cease their study medication can choose to restart it again at any time. Under such circumstances the investigators will ensure that participants who re-start their trial medication have adequate fresh supplies.

# 8. SAFETY

### 8.1 Safety monitoring

The participant, the general practitioner and cardiologist will be encouraged to report any possible adverse events during the course of the trial.

If conditions occur that may cause renal impairment during study conduct, physicians are encouraged to closely monitor renal function following current treatment guidelines. Examples are the administration of nephrotoxic drugs or severe diarrhoea. Management of renal impairment may include (temporary) discontinuation of trial medication. This is to the discretion of the treating physician and/or investigator.

### 8.2 Site monitoring

During the trial, participating sites will be monitored to ensure completeness of patient records, accuracy of entries on the electronic case report forms, adherence to the protocol and handling of trial medication in accordance to the guidelines for good clinical practice. Execution of this monitoring will be organised by dedicated monitors.

### 8.3 (Serious) Adverse events and suspected, unexpected severe adverse reactions.

Monitoring of sudden unexpected serious adverse reactions (SUSARS) and serious adverse events (SAE) will be performed.

#### 8.3.1 Adverse events (AEs)

Adverse Events are defined as any undesirable experience (either a medical occurrence or worsening of pre-existing medical condition) occurring to a subject during the study, whether or not considered related to the trial medication. Based on the broad experience with colchicine, adverse event reporting will only be mandatory for a subset of events:

- Non-serious adverse reactions leading to discontinuation of trial medication -(adverse drug reaction).
- Non-serious AEs of special interest, in the light of colchicine safety:
  - Myopathy
  - Neuropathy
  - Myositis
  - Neutropenia

#### 8.3.2 Serious adverse events (SAEs)

An SAE is any untoward medical occurrence that:

- results in death
- is life-threatening or results in persistent or significant disability/incapacity,
- requires inpatient hospitalisation or causes prolongation of existing hospitalisation,
- results in persistent or significant disability/incapacity or
- Is a congenital anomaly/birth defect.

SAE reporting is performed per ICH-GCP by the investigators within 24 hours of knowledge by the investigator team, to the project office. Expedited reporting to the authorities will be done by the respective project offices according to the local applicable guidelines.

#### 8.3.3 Suspected unexpected serious adverse reactions (SUSARs)

Adverse reactions are all untoward and unintended responses to an investigational product related to any dose administered.

Adverse reactions are SUSARs if the following three conditions are met:

1. The event must be serious
2. There must be a certain degree of probability that the event is a harmful and an undesirable reaction to the investigational product, regardless of the administered dose (as assed by the principal investigator)
3. The adverse reaction must be unexpected, that is to say, the nature and severity of the adverse reaction are not in agreement with the product information as recorded in the summary of product characteristics.

The expedited reporting will occur not later than 15 days after first knowledge of the adverse reactions in the local applicable system. For fatal or life-threatening cases the term will be maximal seven days for a preliminary report with another eight days for completion of the report.

# 9. STATISTICAL ANALYSIS

Central tendencies and distribution of continuous parameters are displayed using mean or median with standard deviation or 25th and 75th percentile in case of normally and non-normally distributed variables respectively. Categorical variables are presented as proportions. Paired differences of parameters are evaluated using the mean or median of the differences and the corresponding 95% confidence interval (CI). A Hodges–Lehmann estimator is used to provide a pseudo-median of the differences in non-parametric distributions. Formal hypothesis testing will be done using a paired sample T – test for normally distributed differences and the Wilcoxon Signed Rank Test with continuity correction for non-normally distributed differences. The linear relationship of two continuous parameters will be calculated using the Spearman's rank correlation coefficient.

# 10. ETHICAL CONSIDERATIONS

### 10.1 Regulation statement

The study will be conducted according to the current Declaration of Helsinki, the Good Clinical Practice guidelines and according to the local guidelines, regulations and acts. Documented approval of appropriate Ethics Committee will be obtained prior to start of the study.

### 10.2 Recruitment and consent

All relevant information on the study will be summarized in an integrated subject information sheet and informed consent form. The study is explained by the investigator or designee based on this document prior to entry to the study. Subject will have enough time to decide if he/she wants to participate, to ask questions and will be informed about the right to withdraw from the study at any time without having to provide reason. The informed consent will be revised when new important information comes available. No study-specific procedures will be conducted prior to receiving informed consent.

# 11. ADMINISTRATIVE ASPECTS, MONITORING AND PUBLICATION

### 11.1 Handling and storage of data and documents

Relevant patient data and documents will be electronically captured in a dedicated database. Data will be collected by study personnel in participating sites. Regulatory documents will be archived in a central trial master file and investigator site files.

All data bases are password protected. All hard copies and electronic records will be kept for 15 years after conclusion of the study.

### 11.2 Amendments

All substantial amendments will be approved by the Ethics and Regulatory committees.

# 12. RISK ANALYSIS

No structured risk analysis including mechanism of action, pharmacokinetic considerations and management of effect is described in this protocol as colchicine is a registered product.

# REFERENCES

1. World Health Organisation (WHO). Top 10 causes of death worldwide. http://www.who.int/mediacentre/factsheets/fs310/en/. Published 2018. Accessed April 23, 2019.

2. De Hartstichting. Hart en vaatziekten in Nederland. https://www.hartstichting.nl/downloads/cijfers/hart-en-vaatziekten-in-Nederland-2015. Published 2015.

3. Davies MJ. Stability and instability: two faces of coronary atherosclerosis. The Paul Dudley White Lecture 1995. *Circulation*. 1996;94(8):2013-2020.

4. Ross R. Atherosclerosis — An Inflammatory Disease. *N Engl J Med*. 1999;340(2):115-126. doi:10.1056/NEJM199901143400207

5. Libby P, Ridker PM, Hansson GK. Progress and challenges in translating the biology of atherosclerosis. *Nature*. 2011;473(7347):317-325.

6. Ridker PM, Luscher TF. Anti-inflammatory therapies for cardiovascular disease. *Eur Heart J*. 2014;35(27):1782-1791. doi:10.1093/eurheartj/ehu203 [doi]

7. Nuki G. Colchicine: its mechanism of action and efficacy in crystal-induced inflammation. *Curr Rheumatol Rep*. 2008;10(3):218-227.

8. Crittenden DB, Lehmann RA, Schneck L, et al. Colchicine use is associated with decreased prevalence of myocardial infarction in patients with gout. *J Rheumatol*. 2012;39(7):1458-1464. doi:10.3899/jrheum.111533 [doi]

9. Solomon DH, Liu CC, Kuo IH, Zak A, Kim SC. Effects of colchicine on risk of cardiovascular events and mortality among patients with gout: a cohort study using electronic medical records linked with Medicare claims. *Ann Rheum Dis*. November 2015. doi:annrheumdis-2015-207984 [pii]

10. Nidorf SM, Eikelboom JW, Budgeon CA, Thompson PL. Low-dose colchicine for secondary prevention of cardiovascular disease. *J Am Coll Cardiol*. 2013;61(4):404-410.

11. Niel E, Scherrmann J-M. Colchicine today. *Jt Bone Spine*. 2006;73(6):672-678.

12. Terkeltaub RA. Colchicine update: 2008. In: *Seminars in Arthritis and Rheumatism*. Vol 38. Elsevier; 2009:411-419.

13. Solak Y, Atalay H, Biyik Z, et al. Colchicine Toxicity in End-Stage Renal Disease Patients. *Am J Ther*. 2014;21(6):e189-e195. doi:10.1097/MJT.0b013e31825a364a

14. Lehmann EL. *Nonparametrics : Statistical Methods Based on Ranks, Revised*. Springer-Verlag New York; 1998.

15. Inc TPUSA. *Full Prescription Information for Colcrys (Colchicine, USP). Retrived from Http://Www.Accessdata.Fda.Gov/Drugsatfda_docs/Label/2009/022351lbl.Pdf*.; 2009.

16. Khanna D, Fitzgerald JD, Khanna PP, et al. 2012 American College of Rheumatology guidelines for management of gout. Part 1: systematic nonpharmacologic and pharmacologic therapeutic approaches to hyperuricemia. *Arthritis Care Res (Hoboken)*. 2012;64(10):1431-1446.
